# Baseline and early changes in laboratory parameters predict disease severity and fatal outcomes in COVID-19 patients

**DOI:** 10.3389/fpubh.2023.1252358

**Published:** 2023-12-13

**Authors:** Addisu Gize, Yerega Belete, Melkayehu Kassa, Wondewosen Tsegaye, Gadissa Bedada Hundie, Birhan Mesele Belete, Mahteme Bekele, Berhan Ababaw, Yosef Tadesse, Bereket Fantahun, Sisay Sirgu, Solomon Ali, Anteneh Mehari Tizazu

**Affiliations:** ^1^School of Medicine, St. Paul’s Hospital Millennium Medical College, Addis Ababa, Ethiopia; ^2^CIHLMU Center for International Health, LMU University Hospital, LMU Munich, Germany; ^3^Department of Internal Medicine, School of Medicine, College of Health Science and Medicine, Wollo University, Dessie, Ethiopia

**Keywords:** COVID-19, mortality, severity, laboratory parameters, resource-limited countries

## Abstract

**Introduction:**

Coronavirus disease 2019 (COVID-19) has become the worst catastrophe of the twenty-first century and has led to the death of more than 6.9 million individuals across the globe. Despite the growing knowledge of the clinicopathological features of COVID-19, the correlation between baseline and early changes in the laboratory parameters and the clinical outcomes of patients is not entirely understood.

**Methods:**

Here, we conducted a time series cross-sectional study aimed at assessing different measured parameters and socio-demographic factors that are associated with disease severity and the outcome of the disease in 268 PCR-confirmed COVID-19 Patients.

**Results:**

We found COVID-19 patients who died had a median age of 61 years (IQR, 50 y – 70 y), which is significantly higher (*p* < 0.05) compared to those who survived and had a median age of 54 years (IQR, 42y – 65y). The median RBC count of COVID-19 survivors was 4.9 × 10^6^/μL (IQR 4.3 × 10^6^/μL – 5.2 × 10^6^/μL) which is higher (*p* < 0.05) compared to those who died 4.4 × 10^6^/μL (3.82 × 10^6^/μL – 5.02 × 10^6^/μL). Similarly, COVID-19 survivors had significantly (*p* < 0.05) higher lymphocyte and monocyte percentages compared to those who died. One important result we found was that COVID-19 patients who presented with severe/critical cases at the time of first admission but managed to survive had a lower percentage of neutrophil, neutrophil to lymphocyte ratio, higher lymphocyte and monocyte percentages, and RBC count compared to those who died.

**Conclusion:**

To conclude here, we showed that simple laboratory parameters can be used to predict severity and outcome in COVID-19 patients. As these parameters are simple, inexpensive, and radially available in most resource-limited countries, they can be extrapolated to future viral epidemics or pandemics to allocate resources to particular patients.

## Introduction

Coronavirus disease 2019 (COVID-19) is caused by the novel severe acute respiratory syndrome coronavirus 2 (SARS-CoV-2) and has become the worst catastrophe of the twenty-first century. As of 19 April 2023, more than 763 million confirmed cases and more than 6.9 million deaths have been reported (1). COVID-19 has a diverse spectrum of clinical manifestations ranging from asymptomatic cases to more severe and critical cases. Patients’ factors including clinical, immunological, hematological, and demographic characteristics influence the course of the disease (2, 3). Our overall knowledge and understanding of the basic pathophysiology of COVID-19 from the start of infection up to clinical manifestation have evolved (4). Likewise, the development of vaccines has played a pivotal role in controlling and altering the course of the pandemic saving millions of lives globally (5, 6).

On March 13, 2020, the first case of COVID-19 was reported in Addis Ababa, Ethiopia. Since then, as of 19 April 2023, there have been more than half a million confirmed cases, and 7,574 deaths have been registered (7). Simple parameters that are associated with the severity of COVID-19 and death or improvement of the disease have implications in early diagnosis and in allocating resources to specific groups of patients that tend to progress to develop into severe cases and further die of the disease (8). Factors like the presence of comorbidity, sex, and age influence the severity of COVID-19 and the outcome of the disease. Patients can also have a post-infectious hyper-inflammatory disease and long-lasting (>2 months) COVID-19 symptoms which are termed as long COVID-19 (9). Besides this, the course of the disease could change after hospitalization, that is, individuals presented with mild or moderate initial symptoms can suffer from more severe symptoms affecting multiple organs of the patient (10).

In the past century, increased global travel, urbanization, and changes in the natural environment have increased the likelihood of the occurrence of pandemics (11). The pattern of viral outbreaks differs in many ways, like the mortality rate associated with the virus, individuals at risk of infection, and viral transmissibility rate widely differ (12). For instance, COVID-19 illness in cancer patients is associated with a significant rate of hospitalization and severe outcomes. Additionally, a particular cancer treatment with immune checkpoint inhibitors also predict severe disease outcome (13). Similarly, old age, male sex, and comorbidities like dementia and cardiovascular and lung diseases were shown to predict COVID-19 infection and death (14). On the other hand, simple laboratory parameters like low lymphocyte count and clinical parameters like a high median Radiographic Assessment of Lung Edema (RALE) were associated with mortality due to COVID-19 (15). Laboratory parameters like elevated CRP and IL-6 predicted COVID-19 disease severity and the need for mechanical ventilation in COVID-19 patients (16). The level of IL-6 has also been reported to be elevated in complicated COVID-19 patients (17). Similarly, the plasma level of IL-2, IL-7, IL-10, and TNF-α increased in ICU COVID-19 patients compared to non-ICU COVID-19 patients (18). Thus, socio-demographic determinants and simple clinical and laboratory parameters that are associated with severity and death among COVID-19 patients would be important to manage the patients as early as possible. These parameters can be extrapolated to future viral outbreaks of similar types.

Here, we sought to evaluate the prognostic use of socio-demographic data, clinical data, and laboratory parameters at the time of admission and during patients’ hospital stays that distinguished survivors from non-survivors of COVID-19. We tried to assess the dynamics of the measured parameters to characterize the whole course of the disease and the final outcome of the patient. Our overall intent was to identify simple and inexpensive prognostic parameters from admission to discharge or death that influenced the outcome of COVID-19 patients. This is especially important in allocating resources efficiently in resource-limited countries like Ethiopia to decrease mortality in COVID-19 patients; also, these parameters can be employed in the future if a pandemic or epidemic of a similar disease occurs.

## Materials and methods

### Study setting

The study was conducted at St. Paul’s Hospital Millennium Medical College (SPHMMC) which is located in Addis Ababa, Ethiopia, and was one of the main COVID-19 isolation and treatment centers in the country. It is a referral-specialized hospital, estimated to serve a total population of more than 5 million people. Patients from Addis Ababa city and rural areas around the city suspected of having COVID-19 were referred to the center. The COVID-19 isolation and treatment center had 260 beds delegated for COVID-19 patients. From 1 October 2020 to 16 September 2021, we used the national Ethiopian Public Health Guideline (EPHG) (19) and enrolled adult patients 18 years old and above who met the eligibility criteria for the study.

### Study design

A time series cross-sectional study was conducted for 1 year from 1 October 2020 to 16 September 2021.

### Study participants

All adult patients (18 years old and above) having any acute respiratory illness (e.g., runny nose and sore throat) and at least one of the following symptoms: fever, cough, and shortness of breath were used as a source population of the study. All suspected COVID-19 patients who tested positive for SARS-CoV-2 by real-time PCR (RT-PCR) test and met the eligibility criteria were enrolled in the study. Our participants were all recruited from the local population and we did not encounter international patients that took the COVID-19 vaccine during the study period. Patients who were unwilling to participate and were previously known to have COVID-19 were excluded from the study. Once the study participants were enrolled in the study, they were followed up until discharge or death. All patients were isolated and stayed in the hospital until they tested negative for the SARS-CoV-2 by RT-PCR test. On average, mild/moderate COVID-19 patients stayed for 2 weeks, whereas those severe/critical patients stayed an average of 6 weeks and were followed up until discharge or death. Those participants who were discharged or died before the second set of measurements were taken were excluded from the study. COVID-19 patients enrolled in this study were diagnosed based on WHO interim guidance into mild (symptomatic patients without evidence of viral pneumonia or hypoxia), moderate (patients with signs of pneumonia and SpO2 ≥ 90% on room air), severe (signs of pneumonia plus one of the following: respiratory rate > 30 breaths/min; severe respiratory distress; or SpO2 < 90% on room air), and critical cases (patients that develop Acute Respiratory Distress Syndrome (ARDS), septic shock or multi-organ dysfunction) (20).

### Sample size and sampling procedure

**Figure 1:**
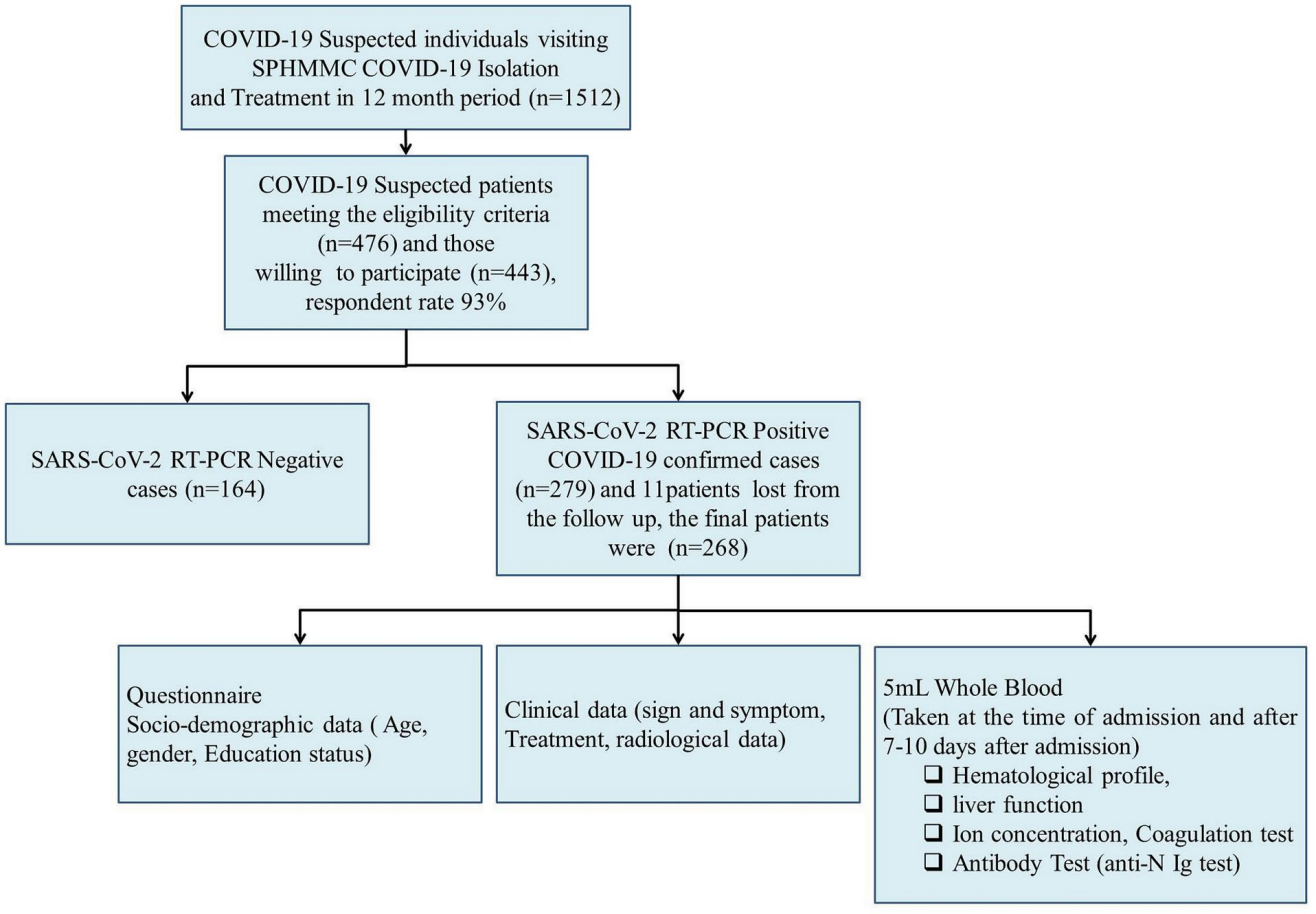
Conceptual framework of the study. We consider a broad conceptual framework spanning aspects of the socio-demographic, clinical data, and laboratory parameters associated with disease severity and final outcome of COVID-19 patients.

The study was conducted during the peak period of the first COVID-19 wave and our institute allocated a total of 260 beds for COVID-19 patient isolation and treatment. Thus, we found the convenient sample technique a more appropriate method for recruiting study participants. A consecutive convenient sampling technique was used to recruit study participants until the end of the study period. During the study period, a total of 1,512 COVID-19-suspected patients visited the SPHMMC COVID-19 isolation and treatment center. Of the suspected patients, 476 met the eligible criteria and 443 were willing to participate with a response rate of 93% (Figure 1). Oropharyngeal (OP) and/or nasopharyngeal (NP) samples were tested for SARS-CoV-2 RNA by RT-PCR and we excluded 164 patients who tested negative, and 279 patients tested positive of whom 11 participants were lost from the follow-up. Finally, we managed to include 268 study participants, and demographic, clinical, and laboratory parameters were recorded and assessed for potential predictors (Figure 1). Once the patients were admitted, blood samples were collected within 24 h of the time of admission, and a second blood sample was collected between 7 and 10 days after admission. COVID-19 patients were assessed for disease severity at admission and followed up till death or discharge from the hospital. We took samples proportionally throughout the year and the effect of a particular SARS-CoV-2 variant would not significantly affect the whole sample.

### Socio-demographic and clinical data

A pre-tested questionnaire and checklist were used to collect the study participants’ socio-demographic data, clinical data, and laboratory test results. The data were collected by trained physicians and laboratory technologists and reviewed by other investigators. Socio-demographic data were collected at the time of admission and clinical data were taken at the time of admission and during the patients’ hospital stay. Any information that could identify individual participants during or after data collection was kept private. Accordingly, patient clinical data on initial clinical presentation, comorbidities, and relevant treatment and clinical outcomes for all patients presented with acute respiratory symptoms or influenza-like illness symptoms were recorded. Oropharyngeal (OP) and/or nasopharyngeal (NP) swabs and a blood sample were collected within 24 h of patient admission and transported to SPHMMC laboratory for analysis and a second blood sample was collected between 7 and 10 days after admission for further assessment of patient progress. Collected data and measured parameters were pooled and cleaned at a global level for further analysis. A comparison of measured parameters was made between different groups like survivors vs. non-survivors and mild/moderate vs. severe/critical cases.

### RNA extraction and RT-PCR analysis

Oropharyngeal (OP) and/or nasopharyngeal (NP) swabs were collected according to the Ethiopian Public Health Institute Guideline (EPHI) (19). The samples were transported to the SPHMMC COVID-19 laboratory in VTM (viral transport media) (in a cold chain of 2-8°C) for RT-PCR analysis of SARS-CoV-2. Two hundred microliters (200 μL) of the NP or OP swab were mixed with 50 μL proteinase K and 200 μL lysis buffer that contains a guanidinium-based inactivating agent, and then viral RNA was extracted using a nucleic acid isolation Kit (Da’an Gene Corporation). Then, viral RNA was eluted with 60 μL elution buffer, and an RT-PCR reagent (Da’an Gene Corporation) was employed for SARS-CoV-2 detection. Two PCR primer and probe sets, which target the open reading frame 1ab (ORF 1ab/N) (FAM reporter) and nucleocapsid protein genes and VIC reporter genes, were added to the same reaction mixture. In each run, positive and negative controls were included. Samples were considered to be positive when both sets gave a reliable signal (≤40 CT value). We used primers and sequence-specific fluorescence probes designed and tailored to the highly conservative region of the COVID-19 genome. The important steps for RT-PCR amplifications took place at 50°C for 15 min, 95°C for 3 min, followed by 45 cycles at 95°C for 15 s and 60°C for 30s. All three cycles were accomplished within an hour and 35 min of the reaction being started.

### Anti-N Ig antibody test

The purpose of this antibody test is to confirm the participants’ immune response to the natural history of SARS-CoV-2 infection. The test was performed by the Elecsys anti-SARS-CoV-2 assay (Elecsys Anti-N; Roche Diagnostics, International Ltd., Rotkreuz, Switzerland) which can indicate the existence of anti-nucleocapsid antibodies during natural infection. The cut of index values below 1.0 (COI < 1.0) was interpreted as non-reactive or negative for anti-SARS-CoV-2 antibodies, while the cut of index values greater than or equal to 1.0 (COI ≥ 1.0) was interpreted as reactive or positive.

### Hematology tests

Fresh (<4 h from collection) whole blood samples (8–10 mL) were collected aseptically from each study subject using di-potassium EDTA–anti-coagulated vacutainer tubes (Becton Dickinson) within 24 h and 7–10 days after patient admission. The sample was analyzed using Beckman Coulter DxH 800 Hematology Analyzer (California, United States). The DxH 800 hematology analyzer measures 28 different hematological parameters like size, shape, and structure of the cell that can be recorded.

### Biochemical and enzymatic tests

Serum/Plasma from coagulated blood was used for enzymatic and biochemical tests for liver and renal function assessment using Cobas C 501 Chemistry Analyzer (Roche) at the clinical chemistry laboratory. The instrument is fully automated and can analyze up to 60 assays like Glucose (Glu), Hemoglobin A1C (HBA1C), and Alanine Aminotransferase (ALT). The plasma sample was used for the quantitative determination of total cholesterol (CHOL), triglycerides (TG), high-density level cholesterol (HDL-C), and low-density level cholesterol (LDL-C), using *Roche Cobas* C311 (Roche/Hitachi).

### Electrolyte tests

The ion concentration was determined using the Roche-Cobas-C311 (Roche/Hitachi) clinical chemistry analyzer. The machine uses an ion-selective electrode (ISE) system to measure the serum’s sodium, potassium, and chloride levels. The ISE uses the unique properties of certain membrane materials to develop an electrical potential [electromotive force (EMF)] for the measurements of ions in the solution.

### Coagulation test

We used the Stago STA Compact Coagulation Analyzer (Diagnostica Stago), a robust and practical benchtop coagulation system to determine the different coagulation parameters. We measured prothrombin time (PT), a test that measures how long it takes for a clot to form in a blood sample, normally resulting in 10 to 14 s. The partial thromboplastin time [PTT; also known as activated partial thromboplastin time (aPTT)] was used to evaluate a person’s ability to appropriately form blood clots. It measures the number of seconds it takes for a clot to form in a sample of blood after reagents are added, normally resulting in 26–35 s, and an INR (international normalized ratio) is a type of calculation based on PT test results. In healthy people, an INR of 1.1 AU (Arbitrary Unit) or below is considered normal. An INR range of 2.0 AU to 3.0 AU is generally an effective therapeutic range for people taking warfarin for certain disorders.

### Data quality assurance

All procedures and steps were pre-tested and proper amendments were taken before the actual data collection. For laboratory analysis, pre-analytical, analytical, and post-analytical stages of the laboratory work were done as per the Standard Operating Procedures (SOPs) of the lab to maximize the quality of collected data. Additionally, positive and negative controls were used in the laboratory analysis during each run. Quality controls were performed daily and after every additional calibration. We put effort into minimizing the missed data by simplifying the questionnaires and explaining the whole purpose of the study to increase the accuracy of the data.

### Data entry and analysis

The data were pooled together and entered into an Excel spreadsheet and subjects with incomplete information (those lost from follow-up) were removed from the analysis. The distribution of the data was checked using the Shapiro–Wilk test using R software (V 4.2.2, R Core Team, 2021), and means and Standard Deviation (SD) or median and interquartile ranges were used to describe contentious data with or without normal distribution, respectively, and the percentage was used for a categorical data set. A statistical test was done using SPSS version 23.0 (SPSS, Inc., Chicago, IL). For univariate analysis, Mann–Whitney U test, Chi-square, and Fisher’s Exact test were used to compare the two groups (alive vs. deceased as well as mild/moderate vs. severe/critical cases). The multicollinearity between the independent variables was checked using a Variance Inflation Factor (VIF), and a VIF of less than 3 was used for logistic regression. Independent variables with a *p*-value < 0.3 during univariate analysis were selected for the final logistic regression. The graphs were generated using the R 4.2.2 package (RcmdrPlugin.KMggplot2) (R Core Team, 2021). A 95% CI and *p* ≤ 0.05 was considered statistically significant.

### Ethics approval and consent to participate

The detailed study protocol was reviewed and clearance to carry out this study was obtained from the institutional review board (IRB) of St. Paul’s Hospital Millennium Medical College, Addis Ababa, Ethiopia. The objectives of the study were explained to all patients using the local language and patients were informed that they have the right not to participate or withdraw at any time and any stage from the study. The patients were also informed that their willingness to or not to participate does not affect their treatment at the hospital. The consent form was written in English and Amharic (the local language) so that the patient had full information about the study. For those illiterate patients, the full information and objectives of the study were explained verbally and written informed consent was taken from each participant. A unique code was given to participants and any information concerning the patients was kept confidential and the specimens collected from the patients were analyzed for the intended purpose only. Positive patient results were communicated timeously with the medical doctors who were working at a treatment center for better management.

## Results

### Study subjects

A total of 443 adult patients showing signs and symptoms of COVID-19 (who met the eligibility criteria and were willing to participate) were isolated at St. Paul Hospital’s Millennium Medical College COVID-19 Center at the peak period of transmission from 1-Oct-2020 to 16-Sep-2021. After excluding 168 patients who tested negative for SARS-CoV-2 RNA by RT-PCR, 268 patients were enrolled for the final analysis (Table 1). The mortality rate among the participants was 21.6% (58/268). The median age of the participant was 56 years (IQR 44y – 66.25y) and the mean age of the participant was 55.5 years (SD = 15.3y, min 20y and max 98y). Of the total participants, women comprised 37.3% (*n* = 100) and men accounted for 62.7% (*n* = 168). Participants from the urban area were 82.2% (*n* = 222) and those that came from the rural part of the city were 17.8% (*n* = 46). Of the different symptoms of COVID-19, most participants presented with cough (91%; *n* = 244), short breath (92.2%; *n* = 247), and loss of appetite (85.4%; *n* = 229). We found that the age of the participant at the time of admission significantly (*p* < 0.05) affected the final outcome of the COVID-19 patients. Those COVID-19 patients who died had a significantly higher (*p* < 0.05) median age of 61 years (50 y – 70 y) at the time of admission compared to those who survived and had a median age of 54 years (IQR 42y – 65y) (Table 1), which align with another study (21). On the other hand, we found no significant association (*p* > 0.05) with gender, presence of comorbidity, marital status, education status, and clinical symptoms at the time of admission on the final outcome of the participants (Table 1). There was also no significant association (*p* > 0.05) between a particular comorbidity with the final outcome of COVID-19 patients (Supplementary Table S1). However, we observed that even if some of the COVID-19 patients who died were free of any disease, they were relatively old [X, IQR (60 (56–70))] compared to those who survived and were free of the disease [X, IQR (51 (38–60))] (*p* < 0.05) (Supplementary Table S2) which could explain our loss of association between comorbidity with fatal outcome of COVID-19, and age was one of the risk factors of COVID-19’s fatal outcome.

**Table 1:** Socio-demographic characteristics of the study participants (*n* = 268).

Characteristics	Death (*N* = 58)	Alive (*N* = 210)	χ^2^	*p*-value
Age, median (IQR)	61 (50–70)	54 (42–65)	4,481	0.00206
Gender, female *n* (%)	20 (20)	80 (80)	0.253	0.614
**Resident**				
Urban	46 (20.7)	176 (79.3)	0.328	0.566
Rural	12 (26)	34 (74)		
**Marital status**				
Single	9 (25)	27(75)	4.77	
Married	37 (20)	148 (80)		
Divorce	2 (18.2)	9 (81.8)		0.31
Widowed	8 (24.2)	25 (75.8)		
Separated	2 (66.7)	1 (33.3)		
**Education status**				
Illiterate	12 (24.5)	37 (75.5)	1.55	0.67
Primary	11 (23.4)	36 (76.6)		
Secondary	19 (20)	76 (80)		
College and above	16 (20.7)	61 (79.3)		
**BCG vaccination**				
No	41 (24.4)	127 (75.6)	2.53	0.111
Yes	17 (17)	83 (83)		
**Comorbidity**				
No	28 (22.7)	95 (77.3)	0.444	0.505
Yes	30 (20.6)	115 (79.4)		
**Behavioral characters**				
History of alcohol Yes *n* (%)	8 (19)	34 (81)	0.068	0.793
History of cigarette Yes *n* (%)	1 (11.1)	8 (88.9)	1.62	0.444
History of khat Yes *n* (%)	3 (21.4)	11 (78.6)	0.0028	0.957
**Clinical symptoms**				
Cough, Yes *n* (%)	52 (21.3)	192 (78.7)	0.954	0.328
Sneezing, Yes *n* (%)	5 (21.7)	18 (78.3)	0.011	0.914
Short Breath, Yes *n* (%)	50 (20.2)	197 (79.8)	0.906	0.341
Headache, Yes *n* (%)	37 (19.8)	150 (80.2)	0.424	0.514
Sore Throat, Yes *n* (%)	17 (18.3)	76 (81.7)	0.672	0.412
Runny Nose, Yes *n* (%)	2 (11.1)	16 (88.9)	1.07	0.301
GI symptoms, Yes *n* (%)	22 (20.2)	87 (79.8)	0.016	0.899
Ansomia, Yes *n* (%)	10 (14.5)	59 (85.5)	2.27	0.131
Loss Appetite, Yes *n* (%)	47 (20.5)	182 (79.5)	0.092	0.761
Loss of Test, Yes *n* (%)	10 (13.9)	62 (86.1)	3.02	0.081

*Khat is a stimulant that is chewed mainly in East Africa, especially in Ethiopia, Djibouti, Somalia, and Eritrea.

### Baseline-measured clinical and laboratory parameters determine the outcome of COVID-19 patients

**Figure 2:**
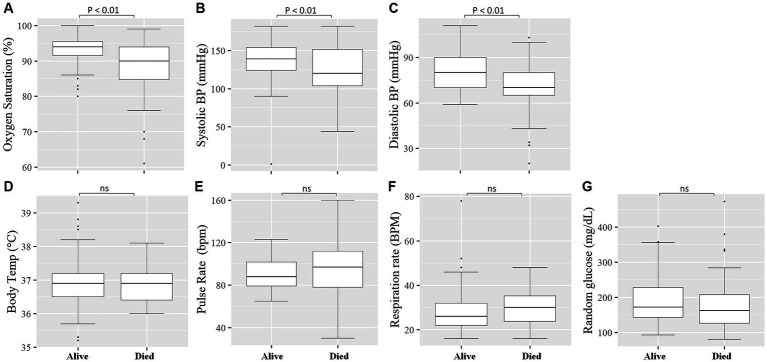
Comparison of vital clinical signs between survivors and deceased COVID-19 patients at the time of admission. The level of Oxygen Saturation (%) **(A)**, Systolic blood pressure (mmHg) **(B)**, and Diastolic blood pressure (mmHg) **(C)** were significantly (*p* < 0.05) lower in deceased COVID-19 patients compared to those that survived, whereas no significant (*p* > 0.05) difference was observed in Body Temperature (°C) **(D)**, Pulse Rate (bpm) (beats per minute) **(E)**, Respiratory Rate (BPM) (breaths per minute) **(F)**, and Random glucose (mg/dL) **(G)** between the two groups.

We measured vital clinical signs at the time of admission, and we found that the level of oxygen saturation (Figure 2A), systolic blood pressure (Figure 2B), and diastolic blood pressure (Figure 2C) were significantly (*p* < 0.05) lower in deceased individuals compared to survivors of COVID-19. On the other hand, body temperature, pulse rate, respiration rate, and random glucose (Figures 2D–G) did not show any significant (*p* > 0.05) difference between the two groups (22). We then looked into the different hematological and clinical parameters measured at the time of admission and assessed their role in determining the outcome of COVID-19 after admission. Univariant analysis of our data showed those COVID-19 patients who survived had significantly (*p* < 0.05) higher lymphocyte and monocyte percentages (Table 2). Others have also shown that COVID-19 patients with low lymphocyte counts have a high mortality rate (15), whereas the percentage of neutrophil and neutrophil-to-lymphocyte ratio (NLR) were significantly lower (*p* < 0.05) in COVID-19 patients who survived compared to those who died (Table 2). The increased NLR level has been linked with COVID-19 disease severity and mortality (23). The RBC count of COVID-19 patients was higher (*p* < 0.05) in patients who survived 4.9 × 10^6^/μL (IQR, 4.3 × 10^6^/μL – 5.2 × 10^6^/μL) compared to those who died 4.4 × 10^6^/μL (IQR, 3.82 × 10^6^/μL – 5.02 × 10^6^/μL) (Table 2). As the normal count of RBC was lower in women (4.2–5.4 × 10^6^/μl) compared to men (4.7–6.1 × 10^6^/ul), we assessed whether the effect of RBC count in COVID-19 patients who survived was still higher than in deceased patients independent of gender. We separately analyzed the data for the RBC level between the two genders and we observed a similar trend, that is, a lower RBC count in deceased COVID-19 patients in both male and female patients (Supplementary Figure S1). Similarly, at the time of admission, the liver function test ALP was significantly lower (*p* < 0.05) in survivors [X (IQR), 70 IU/L (57.25 IU/L–86.75 IU/L)] compared to deceased [X (IQR), 88 IU/L (66.5 IU/L–109.5 IU/L)] patients. In the same manner, at the time of admission, the liver function test GOT, the kidney function test creatinine and urea, and the level of K concentration were lower (*p* < 0.05) in survivors compared to deceased patients (Table 2). A multivariate analysis of measured parameters that have a *p*-value < 0.3 was analyzed, and neutrophil percentage and RBC count were important predictors of COVID-19 outcome (Table 2).

**Table 2:** Univariant and multivariant analysis of different measured parameters (*n* = 268).

Parameters	Alive median (75% IQR)	Dead median (75% IQR)	COR (95%CI)	*p*-value	AOR (95%CI)	*p*-value
**Leukocyte test**						
WBC (10^9^/L)	8.2 (5.9–12.025)	10 (7.1–14.6)	1.02 (0.99–1.06)	0.175		
Lymphocyte (%)	10 (6–17)	5 (3–9)	0.87 (0.81–0.94)	0.0002	1.3 (0.58–3.03)	0.495
Neutrophil (%)	82.4 (73.45–88.2)	88.5 (81–94)	1.07 (1.02–1.12)	0.0021	1.2 (1.04–1.37)	0.014
NLR (AU)	7.7 (4.4–14.4)	15.1 (8.9–31.2)	1.04 (1.02–1.06)	0.0017	0.99 (0.9–1.05)	0.745
Monocyte (%)	6.05 (4.07–8.325)	3.8 (2.475–6.425)	0.86 (0.76–0.98)	0.022	1.3 (0.97–1.7)	0.074
Eosinophil (%)	0.1 (0–0.375)	0.1 (0–0.2)	0.6 (0.29–1.23)	0.163	1.06 (0.17–6.4)	0.942
Basophil (%)	0.2 (0.1–0.4)	0.2 (0.1–0.4)	1.03 (0.81–1.3)	0.795		
**RBC indices**						
RBC (10^6^/μL)	4.8 (4.5–5.225)	4.42 (3.93–4.97)	0.58 (0.38–0.87)	0.008	0.43 (0.2–0.88)	0.023
MCH (pg/Cell)	30.1 (28.9–31.3)	30.1 (28.8–31.5)	0.96 (0.86–1.09)	0.57		
MCHC (g/dL)	34 (33.3–34.6)	33.6 (32–34.4)	0.82 (0.63–1.07)	0.137	1.27 (0.78–2.1)	0.328
RDW (%)	13.7 (13–14.4)	14 (13.6–15.4)	1.1 (0.97–1.3)	0.116	1.1 (0.86–1.4)	0.408
Hgb (g/dL)	14.5 (13.3–15.8)	13.1 (11.7–14.5)	0.86 (0.76–0.97)	0.013		
HCT (%)	42.2 (39.35–45.8)	40.4 (34.4–43.5)	0.94 (0.89–0.98)	0.006		
MCV (fL)	88.2 (85.1–91.3)	89.2 (85–91.9)	1.01 (0.95–1.07)	0.68		
**Coagulation test**						
Platelet (10^3^/μL)	201 (155.8–269.3)	211 (138–259)	1 (1–1)	0.749		
MPV (fL)	8.6 (7.9–9.3)	8.8 (8.4–9.4)	1.38 (0.97–1.9)	0.06	1.7 (1.03–33)	0.036
INR (AU)	1.04 (0.98–1.2)	1.2 (1.09–1.5)	10 (0.96–107.3)	0.053		
PT (sec)	14.1 (13.25–15)	16.6 (14.95–19.8)	1.4 (1.06–1.7)	0.015		
PTT (sec)	29.25 (23.5–33.5)	28.5 (21.75–31.3)	0.98 (0.94–1.1)	0.513		
**Kidney function test**						
Urea (mg/dL)	28.1 (20.2–40.4)	55.1 (28–81.7)	1.01 (1–1.02)	0.0003	1.01 (0.98–1.03)	0.542
Creatinine (mg/dL)	0.79 (0.65–1.01)	1.06 (0.75–1.8)	1.17 (1.03–1.33)	0.012		
**Liver function test**						
ALP (IU/L)	70 (57.25–86.75)	88 (66.5–109.5)	1.01 (1–1.021)	0.002	1.01 (0.9–1.02)	0.641
GOT (IU/L)	36.4 (25.47–53.97)	41.1 (29.7–79.1)	1 (1.001–1.013)	0.031		
GPT (IU/L)	30.05 (18.8–47.45)	31.4 (18.6–49.4)	1 (0.99–1.01)	0.15	1.01 (0.9–1.02)	0.24
DBIL (mg/dL)	0.17 (0.12–0.25)	0.2 (0.14–0.31)	1.8 (0.47–7.34)	0.37		
TBIL (mg/dL)	0.48 (0.342–0.704)	0.47 (0.27–0.71)	1.3 (0.49–3.32)	0.616		
**Ion Con**						
K (mmol/L)	4.4 (3.96–4.7)	4.8 (4.23–5.32)	2.3 (1.4–3.7)	0.0005	2.5 (1.3–4.8)	0.005
Na (mmol/L)	136 (133–140)	137 (134–140)	1.04 (0.99–1.1)	0.101	1.05 (0.96–1.2)	0.31
Cl (mmol/L)	97.5 (94.9–101.2)	97.95 (93–102)	1.02 (0.98–1.07)	0.243		
**Lipid profile**						
HDL (mg/dL)	39.25 (29–44.65)	28.1 (20.8–29.9)	0.9 (0.82–1.01)	0.72		
Cholesterol (mg/dL)	155.35 (146–161)	95.9 (70.6–124.9)	0.96 (0.92–1)	0.68		
TG (mg/dL)	159 (138–272.25)	99 (95–109)	0.95 (0.88–1.02)	0.42		
LDL (mg/dL)	95.3 (72.9–125.3)	32.9 (28–40.5)	1 (1–1)	0.991		

*Multicollinearity was tested between the different independent variables and VIF < 0.3 was taken for multivariant analysis. The VIF among Hemoglobin level, Hematocrit level, and RBC count was VIF > 10, thus only RBC count was taken for multivariant analysis as the lowest *p*-value. Similarly, the VIF > 5 was observed between ALP and GPT and between Na and Cl (AU = Arbitrary Unit).

### The severity of COVID-19 was linked with measured parameters and the final outcome

**Figure 3:**
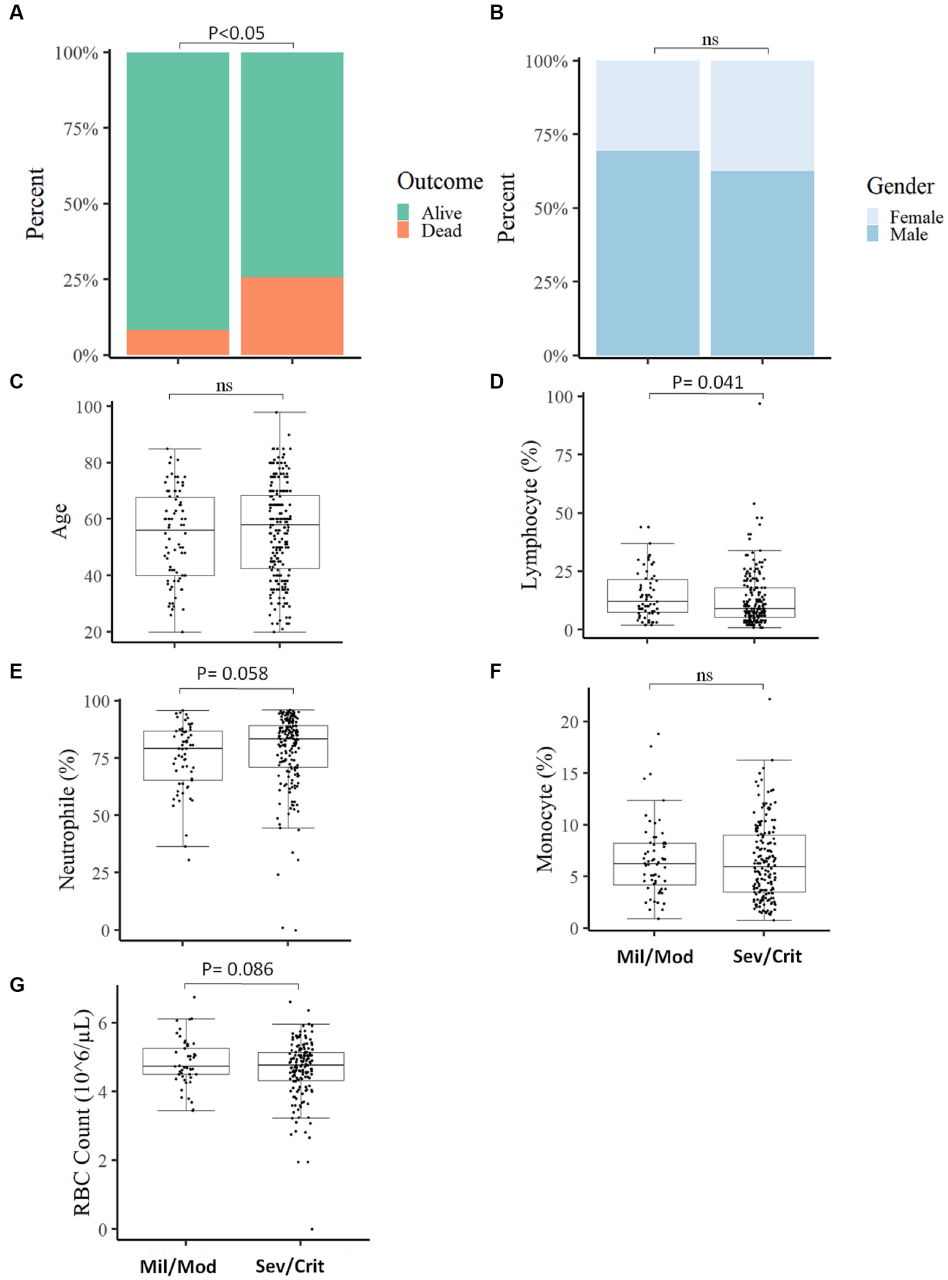
Comparison of baseline measured parameters between Mild/Moderate with Severe/Critical COVID-19 cases. The proportion of individuals deceased was significantly higher in severe/critical cases compared to mild/moderate cases **(A)**. No significant association was observed in disease severity with gender **(B)**, comparison of Age **(C)**, Lymphocyte percentage **(D)**, Neutrophil percentage **(E)**, Monocyte percentage **(F)**, and RBC count **(G)** between mild/moderate and severe/critical case (Mil/Mod = Mild/Moderate, Sev/Crit = Severe/Critical).

We categorized the COVID-19 patients into two groups based on their clinical status at the time of admission and assessed the measured parameters. Of the total COVID-19 patients included in the study, 203 (75.7%) presented with severe/critical COVID-19 and 65 (24.3%) patients manifested mild/moderate COVID-19 symptoms. Depending on the COVID-19 severity, patients were followed up from 2 weeks to 6 weeks. A significant association (*p* < 0.05) was found between the clinical status at the time of admission with the final outcome of the disease, where most deaths (89.6%; *n* = 52) occurred in severe/critical cases compared to 10.4% (*n* = 6) in mild/moderate COVID-19 patients (Figure 3A) which align with another study (24). The clinical status of the participants at the time of admission was not associated (*p* > 0.05) with gender (Figure 3B) and no significant difference (*p* > 0.05) in age between mild/moderate [X, IQR (56y, 40y–67.75y)] compared to severe/critical cases [X, IQR (58y, 42.5y–68.2y)] was observed at the time of admission (Figure 3C). Of the different measured parameters, lymphocyte percentage was significantly higher in mild/moderate [X, IQR (12, 7.5–12%)] compared to severe/critical [X, IQR (9, 5.25–18%)] patients at the time of admission (Figure 3D). On the other hand, other measured parameters like the percentage of neutrophils, the percentage of monocytes, and RBC count did not show a significant difference (*p* > 0.05) between the two groups of patients at the time of admission (Figures 3E–G). We also analyzed the different laboratory parameters in the presence and absence of comorbidity and we found that the presence of comorbidity has no significant (*p* > 0.05) impact on these laboratory parameters and in the final outcome of COVID-19 patients (Supplementary Figure S2).

### Natural antibody production is not associated with COVID-19 outcome

**Figure 4:**
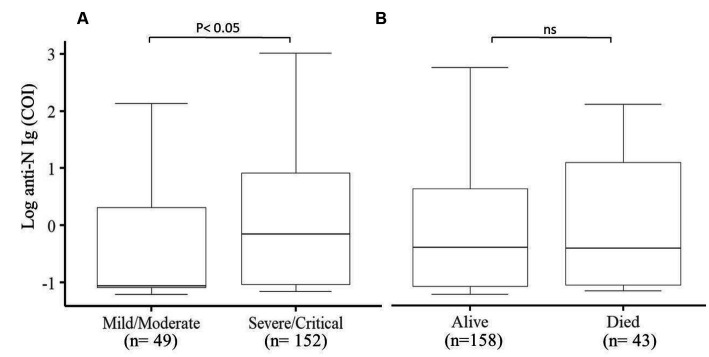
Comparison of the production of natural antibodies in Mild/Moderate cases with Severe/Critical cases and the final COVID-19 outcomes. **(A)** Significantly (*p* < 0.05) higher levels of natural antibodies were produced in severe/critical cases compared to mild/moderate cases and **(B)** comparison between alive and deceased cases. COI (cut of index) (*n* = 201).

We further looked into the natural antibody production of 201 participants and assessed its association with the final outcome and the clinical status of COVID-19 patients. We observed that severe/critical patients produce a much higher level of natural antibodies (*p* < 0.05) compared to mild/moderate COVID-19 patients (Figure 4A), whereas the outcome of COVID-19 patients was not associated with the production of natural antibodies against SARS-CoV-2 (Figure 4B).

### Parameters measured after hospitalization were associated with COVID-19 outcome

**Figure 5:**
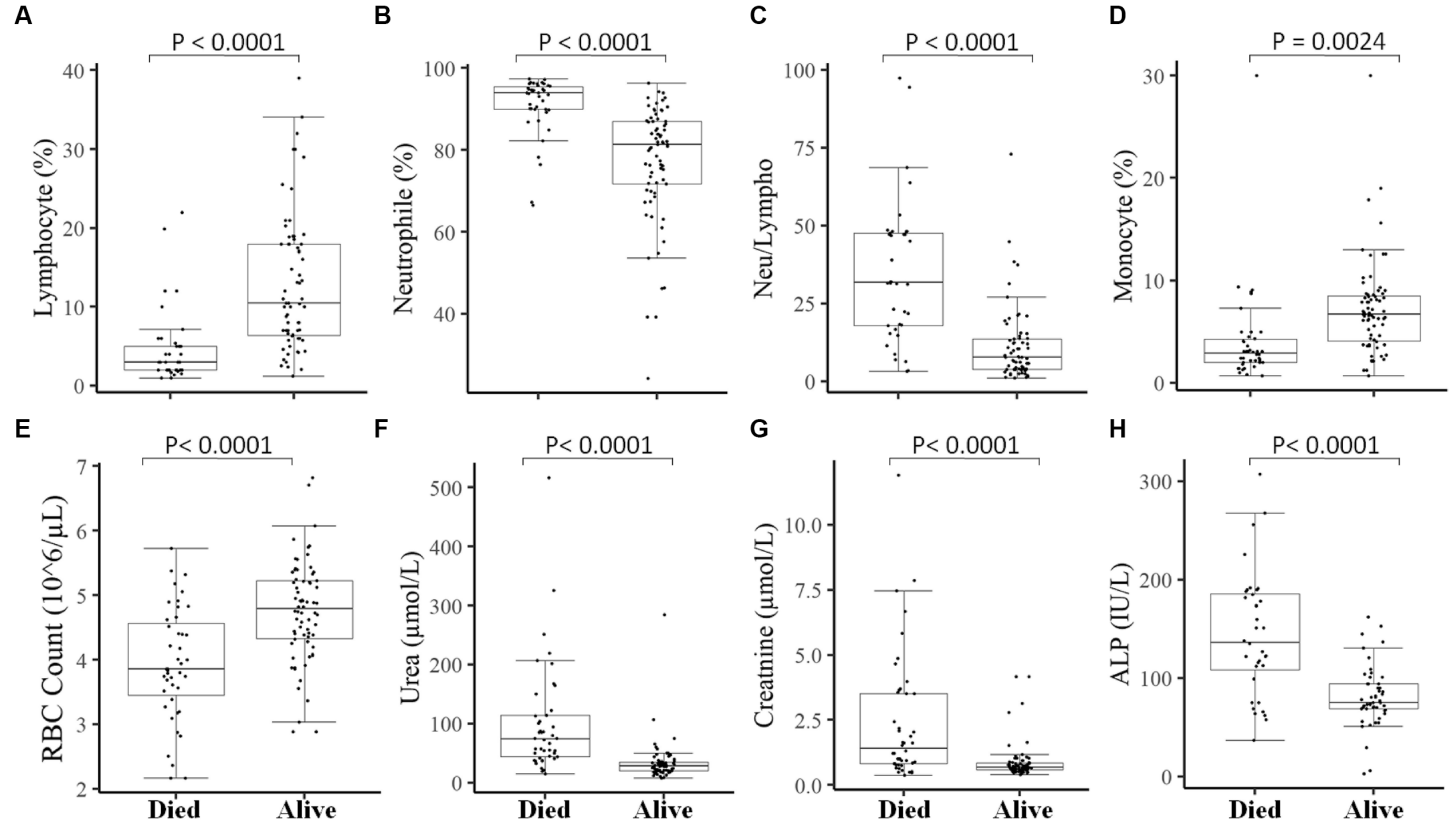
Association of measured parameters after hospitalization of COVID-19 patients with the final outcomes. A significant difference was observed in Lymphocyte percentage **(A)**, Neutrophil percentage **(B)**, Neutrophil-to-lymphocyte ratio **(C)**, Monocyte percentage **(D)**, RBC count **(E)**, Urea **(F)**, Creatinine **(G)**, and ALP **(H)** between those who died vs. those who survived in measurements done after 7–10 days of hospitalization.

We then measured different hematological and clinical parameters after the patients were hospitalized (within 7–10 days) and assessed their association with the patient’s final outcome. COVID-19 patients who survived showed significantly (*p* < 0.05) higher lymphocyte percentage (Figure 5A), monocyte percentage (Figure 5D), and RBC count (Figure 5E) after hospitalization compared to COVID-19 patients who died. On the other hand, COVID-19 patients who survived showed decreased (*p* < 0.05) neutrophil percentage (Figure 5B) and neutrophil-to-lymphocyte ratio (Figure 5C), compared to those deceased COVID-19 patients. Likewise, the kidney function test Urea (Figure 5F), creatinine (Figure 5G), and liver function test ALP (Figure 5H) were significantly lower after hospitalization in COVID-19 patients who survived compared to those who died.

### Maintaining a lower level of different measured parameters helps critical/severe patients to survive COVID-19

**Figure 6:**
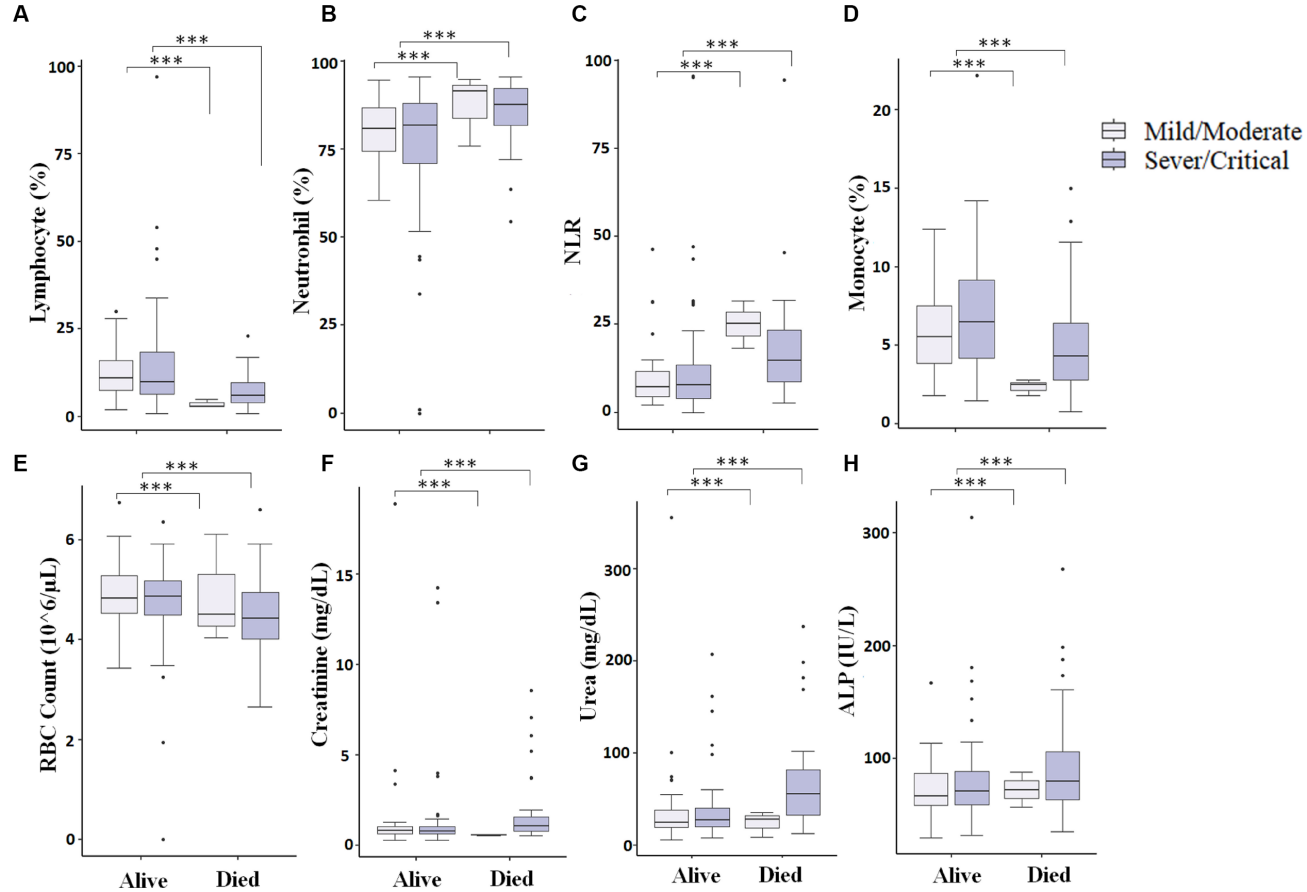
Comparison of different measured parameters at the baseline and after 7–10 days of hospitalization. A significant difference (*p* < 0.05) was observed in Lymphocyte percentage **(A)**, Neutrophil percentage **(B)**, NLR (Neutrophil-to-lymphocyte ratio) **(C)**, Monocyte percentage **(D)**, RBC count **(E)**, Creatinine **(F)**, Urea **(G)**, and ALP **(H)** between those who died vs. those who survived at baseline and after 7–10 days of hospitalization (*** represents a *p* < 0.0001).

We finally looked into patients who were presented as critical/severe cases at the time of admission, and those who were able to survive showed different measured parameters compared to critical/severe patients who died. We observed that critical/severe patients who survived had significantly (*p* < 0.05) increased lymphocyte (%) (Figure 6A), monocyte (%) (Figure 6D), and RBC count (Figure 6E) and significantly (*p* < 0.05) decreased neutrophil (%) (Figure 6B), NLR (Figure 6C), and creatinine, urea, and ALP levels (Figures 6F–H) compared to critical/severe patients who died. This could suggest that favorable outcomes can be achieved by monitoring critically ill COVID-19 patients to maintain measured parameters.

## Discussion

The COVID-19 pandemic has affected all communities across the globe. Even the health systems of developed nations struggled to control the spread of the disease and treat patients during the pandemic (25). Moreover, the disruption of the health system and the overall impact of COVID-19 in developing nations like Ethiopia was also high (26). Understanding the transmission of the virus, pathogenesis, disease course, and, finally, the development of vaccines has halted the pandemic (6, 27). Simple and convenient laboratory parameters that can predict the course of the disease are important to distinguish between patients and give prompt care to those in need. Thus, in this study, we assessed different socio-demographic, clinical, and biological parameters and their role in disease severity and fatal outcomes of COVID-19 patients.

Our result indicates that an increase in age negatively impacts the outcome of COVID-19 patients. An increase in age has been linked with many changes in the body including the deregulation of the immune system, which could make older adults more vulnerable to severe/critical COVID-19 cases (2, 28). Similarly, the vulnerability of older adults could be linked with the pathogenesis of SARS-COV-2, as it uses the angiotensin-converting enzyme 2 (ACE2) to enter into the cell, and the level of ACE2 increases with age (29). While gender was not linked with the severity or mortality of COVID-19 in the present study, others have shown that male patients tend to develop more severe forms of COVID-19 (30).

Our finding showed that vital clinical signs like oxygen saturation, systolic blood pressure, and diastolic blood pressure were significantly lower in deceased COVID-19 patients. Likewise, others also showed decreased oxygen saturation, and diastolic pressure was significantly associated with mortality among COVID-19 patients (22). We found hematological parameters like the decreased lymphocyte percentage and the increase in neutrophil percentage and neutrophil-to-lymphocyte ratio (NLR) in severe COVID-19 patients as well as in those patients with fatal outcomes. This indicates that severe COVID-19 cases tend to develop leukocytosis and lymphocytopenia (31). Similar to our finding, other studies have shown that the NLR value predicts critical illness incidence (32, 33). Likewise, in severe COVID-19 patients, increased neutrophil count has been linked with the development of thrombosis, and post-mortem sample analysis showed high pulmonary infiltration of neutrophils (34, 35). This could suggest the important role of these cells in the pathogenesis of COVID-19 and the role of inflammation in aggravating the disease. Others also showed that increased RDW was observed in severe COVID-19 patients and predicted mortality of patients (36). A Meta-analysis study also showed that baseline parameters like increased WBC count, C-reactive protein, creatine kinase (CK), lactate-dehydrogenase (LDH), D-dimer, and lower absolute lymphocyte count were all associated with a higher mortality rate (37). Overall baseline laboratory investigation can be an important predictor of severity and mortality in COVID-19 patients. The alteration in these parameters could be used as a simple tool for monitoring the patient and allowing timely intervention.

Beyond the most common pulmonary involvement of SARS-CoV-2, different studies have reported its involvement in multiple organs. In this study, we observed increased levels of liver function tests like ALT and GOT in deceased and severe COVID-19 patients (38, 39), which could be linked to the involvement of tissue damage in the liver by SARS-CoV-2 (40). Similarly, the level of kidney function test urea was also higher in severe and deceased COVID-19 patients (41). Direct and indirect mechanisms of kidney damage by SARS-CoV-2 have been implicated. It can directly infect kidney cells using the angiotensin-converting enzyme 2 (ACE2) pathway or an indirect kidney injury could be a result of SARS-CoV-2-driven dysregulation like macrophage activation, lymphopenia, and cytokine storm. Hence, understanding the mechanism of SARS-CoV-2 is important in the therapeutic strategy of COVID-19 patients (42).

The natural antibody has been reported to protect against reinfection from different SARS-COV-2 strains and decrease hospitalization and the severity of the disease (43). We looked into the role of natural antibodies and showed that the level of natural antibodies was not associated with the final outcome of the patients but a significantly higher level of natural antibodies was produced in severe/critical patients compared to mild/moderate patients (44). Others also showed that delayed production of natural antibodies was observed in critically ill COVID-19 patients compared to moderately ill patients. This difference could be linked to the kinetics of antibody production in these patients (45). A different time point data analysis of the production of antibodies in relation to disease severity and the outcome of the disease could reveal the clear role of the natural antibodies in predicting the illness severity and progression of the disease.

One important finding of our work is that severe/critical patients who were able to decrease baseline NLR, ALT, and Urea and increase baseline lymphocyte percentage after hospitalization were able to survive. On the other hand, those COVID-19 patients who were unable to regulate these parameters died of COVID-19 which aligns with other findings (46). Another study showed that decreased lymphocyte count and increased ALT, AST, and CRP were linked with clinical unimprovement of COVID-19 patients in hospitals (47). This indicates that monitoring baseline laboratory parameters after hospitalization could help patients improve their health status and help them to survive.

To conclude, here, we manage to compare measured hematological and biochemical parameters at the time of admission and after admission and found that simple and inexpensive parameters like lymphocyte percentage, NLR, and ALT can help distinguish between severe/critical vs. mild/moderate cases as well as deceased vs. survived patients and could have important prognostic value in identifying patients and help save lives by aiding timely intervention. Even if the COVID-19 pandemic has been well managed at present, there is still a potential risk that different variants of SARS-CoV-2 can evolve, and a new novel respiratory virus can emerge or reemerge and cause a similar form of the disease. Thus our result is especially important in allocating medical resources and will help physicians manage their patients in resource-limited settings. One limitation of our study was a limited sample size due to the inadequate resources we had and the capacity of our COVID-19 center (only 260 beds). The small sample size could be one reason that we were not able to find an association between a particular disease with the fatal outcome of COVID-19 patients, thus we recommend other studies from different COVID-19 centers with large sample sizes to validate our result.

## Data availability statement

The raw data supporting the conclusions of this article will be made available by the authors, without undue reservation.

## Ethics statement

The studies involving humans were approved by Institutional Review Board (IRB) of St. Paul’s Hospital Millennium Medical College, Addis Ababa, Ethiopia. The studies were conducted in accordance with the local legislation and institutional requirements. The participants provided their written informed consent to participate in this study.

## Author contributions

AMT, AG, MK, WT, and SA conceived the study and facilitated the setup of the COVID-19 testing laboratory and diagnosis units. AMT, AG, and WT analyzed, interpreted the data, and drafted the manuscript. AMT, AG, YB, MK, WT, GH, BB, MB, BA, YT, BF, SS, and SA reviewed and edited the manuscript. All authors contributed to the article and approved the submitted version.

## Glossary

ALP: Alkaline phosphatase
AU: Arbitrary unit
Cl: Chloride
DBIL: Direct bilirubin
GOT: Glutamic oxaloacetic transaminase
GPT: Glutamic pyruvic transaminase
Hct: Hematocrit
HDL: High-density lipoprotein
Hgb: Hemoglobin
INR: International normalized ratio
K: Potassium
LDL: Low-density lipoprotein
MCH: Mean cell (corpuscular) hemoglobin
MCHC: Mean cell hemoglobin concentration
MCV: Mean cell (corpuscular) volume
MPV: Mean platelet volume
Na: Sodium
NLR: Neutrophil to lymphocyte ratio
PT: Prothrombin time
PTT: Partial thromboplastin time
RBC: Red blood cell
RDW: Red cell distribution width
TBIL: Total-value bilirubin
TG: Triglycerides
WBC: White blood cell

## References

[1] WHO. WHO Coronavirus (COVID-19) Dashboard. Available at: https://covid19.who.int (Accessed April 25, 2023).

[2] Tizazu AM Mengist HM Demeke G. Aging, inflammaging and immunosenescence as risk factors of severe COVID-19. Immun Ageing. (2022) 19:53. doi: 10.1186/s12979-022-00309-5 36369012 PMC9650172

[3] Fan E Beitler JR Brochard L Calfee CS Ferguson ND Slutsky AS. COVID-19-associated acute respiratory distress syndrome: is a different approach to management warranted? Lancet Respir Med. (2020) 8:816–21. doi: 10.1016/S2213-2600(20)30304-0, PMID: 32645311 PMC7338016

[4] Marik PE Iglesias J Varon J Kory P. A scoping review of the pathophysiology of COVID-19. Int J Immunopathol Pharmacol. (2021) 35:205873842110480. doi: 10.1177/20587384211048026 PMC8477699 34569339

[5] Chen X Huang H Ju J Sun R Zhang J. Impact of vaccination on the COVID-19 pandemic in U.S. states. Sci Rep. (2022) 12:1554. doi: 10.1038/s41598-022-05498-z, PMID: 35091640 PMC8799714

[6] Watson OJ Barnsley G Toor J Hogan AB Winskill P Ghani AC. Global impact of the first year of COVID-19 vaccination: a mathematical modelling study. Lancet Infect Dis. (2022) 22:1293–302. doi: 10.1016/S1473-3099(22)00320-6, PMID: 35753318 PMC9225255

[7] WHO. Ethiopia: WHO Coronavirus Disease (COVID-19) Dashboard With Vaccination Data. Available at: https://covid19.who.int (Accessed April 25, 2023).

[8] Limon-de la Rosa N Cervantes-Alvarez E Méndez-Guerrero O Gutierrez-Gallardo MA Kershenobich D Navarro-Alvarez N. Time-dependent changes of laboratory parameters as independent predictors of all-cause mortality in COVID-19 patients. Biology. (2022) 11:580. doi: 10.3390/biology11040580, PMID: 35453779 PMC9028239

[9] Brodin P. Immune determinants of COVID-19 disease presentation and severity. Nat Med. (2021) 27:28–33. doi: 10.1038/s41591-020-01202-8 33442016

[10] Dennis A Wamil M Alberts J Oben J Cuthbertson DJ Wootton D. Multiorgan impairment in low-risk individuals with post-COVID-19 syndrome: a prospective, community-based study. BMJ Open. (2021) 11:e048391. doi: 10.1136/bmjopen-2020-048391, PMID: 33785495 PMC8727683

[11] Jones KE Patel NG Levy MA Storeygard A Balk D Gittleman JL. Global trends in emerging infectious diseases. Nature. (2008) 451:990–3. doi: 10.1038/nature06536, PMID: 18288193 PMC5960580

[12] Wilder-Smith A. COVID-19 in comparison with other emerging viral diseases: risk of geographic spread via travel. Trop Dis Travel Med Vaccines. (2021) 7:3. doi: 10.1186/s40794-020-00129-9, PMID: 33517914 PMC7847598

[13] Robilotti EV Babady NE Mead PA Rolling T Perez-Johnston R Bernardes M. Determinants of severity in cancer patients with COVID-19 illness. Nat Med. (2020) 26:1218–23. doi: 10.1038/s41591-020-0979-0 32581323 PMC7785283

[14] Najar J Broms R Nistotskaya M Dahlström C. Predictors of COVID-19 outcomes among residents of swedish long-term care facilities–a nationwide study of the year 2020. Am J Geriatr Psychiatry. (2023) 31:456–61. doi: 10.1016/j.jagp.2023.01.027, PMID: 36863972 PMC9907792

[15] Ciceri F Castagna A Rovere-Querini P De Cobelli F Ruggeri A Galli L. Early predictors of clinical outcomes of COVID-19 outbreak in Milan. Italy Clin Immunol. (2020) 217:108509. doi: 10.1016/j.clim.2020.108509, PMID: 32535188 PMC7289745

[16] Herold T Jurinovic V Arnreich C Lipworth BJ Hellmuth JC von Bergwelt-Baildon M. Elevated levels of IL-6 and CRP predict the need for mechanical ventilation in COVID-19. J Allergy Clin Immunol. (2020) 146:128–136.e4. doi: 10.1016/j.jaci.2020.05.008, PMID: 32425269 PMC7233239

[17] Coomes EA Haghbayan H. Interleukin-6 in Covid-19: A systematic review and meta-analysis. Rev Med Virol. (2020) 30:e2141. doi: 10.1002/rmv.2141 32845568 PMC7460877

[18] Huang C Wang Y Li X Ren L Zhao J Hu Y. Clinical features of patients infected with 2019 novel coronavirus in Wuhan. China The Lancet. (2020) 395:497–506. doi: 10.1016/S0140-6736(20)30183-5, PMID: 31986264 PMC7159299

[19] The Federal Ministry of Health (FMOH). National Comprehensive COVID 19 Management Handbook. (2020) Ethiopia, The Federal Ministry of Health.

[20] World Health Organization. Clinical management of COVID-19: interim guidance, 27 May 2020. World Health Organization; (2020), 16, 27–32.

[21] Henkens MTHM Raafs AG Verdonschot JAJ Linschoten M van Smeden M Wang P. Age is the main determinant of COVID-19 related in-hospital mortality with minimal impact of pre-existing comorbidities, a retrospective cohort study. BMC Geriatr. (2022) 22:184. doi: 10.1186/s12877-021-02673-1 35247983 PMC8897728

[22] Ikram AS Pillay S. Admission vital signs as predictors of COVID-19 mortality: a retrospective cross-sectional study. BMC Emerg Med. (2022) 22:68. doi: 10.1186/s12873-022-00631-7, PMID: 35488200 PMC9051839

[23] Ciccullo A Borghetti A Zileri Dal Verme L Tosoni A Lombardi F Garcovich M. Neutrophil-to-lymphocyte ratio and clinical outcome in COVID-19: a report from the Italian front line. Int J Antimicrob Agents. (2020) 56:106017. doi: 10.1016/j.ijantimicag.2020.106017, PMID: 32437920 PMC7211594

[24] Li X Xu S Yu M Wang K Tao Y Zhou Y. Risk factors for severity and mortality in adult COVID-19 inpatients in Wuhan. J Allergy Clin Immunol. (2020) 146:110–8. doi: 10.1016/j.jaci.2020.04.006, PMID: 32294485 PMC7152876

[25] Lupu D Tiganasu R. COVID-19 and the efficiency of health systems in Europe. Heal Econ Rev. (2022) 12:14. doi: 10.1186/s13561-022-00358-y, PMID: 35150372 PMC8841084

[26] Mebratie AD Nega A Gage A Mariam DH Eshetu MK Arsenault C. Effect of the COVID-19 pandemic on health service utilization across regions of Ethiopia: An interrupted time series analysis of health information system data from 2019–2020. PLoS Glob Public Health. (2022) 2:e0000843. doi: 10.1371/journal.pgph.0000843, PMID: 36962800 PMC10021875

[27] Alagoz O Sethi AK Patterson BW Churpek M Alhanaee G Scaria E. The impact of vaccination to control COVID-19 burden in the United States: A simulation modeling approach. PLoS One. (2021) 16:e0254456. doi: 10.1371/journal.pone.0254456, PMID: 34260633 PMC8279349

[28] Starke KR Reissig D Petereit-Haack G Schmauder S Nienhaus A Seidler A. The isolated effect of age on the risk of COVID-19 severe outcomes: a systematic review with meta-analysis. BMJ Glob Health. (2021) 6:e006434. doi: 10.1136/bmjgh-2021-006434, PMID: 34916273 PMC8678541

[29] Bickler SW Cauvi DM Fisch KM Prieto JM Sykes AG Thangarajah H. Extremes of age are associated with differences in the expression of selected pattern recognition receptor genes and ACE2, the receptor for SARS-CoV-2: implications for the epidemiology of COVID-19 disease. BMC Med Genet. (2021) 14:138. doi: 10.1186/s12920-021-00970-7, PMID: 34030677 PMC8142073

[30] Statsenko Y Al Zahmi F Habuza T Almansoori TM Smetanina D Simiyu GL. Impact of age and sex on COVID-19 severity assessed from radiologic and clinical findings. Front Cell Infect Microbiol. (2022) 11:11. doi: 10.3389/fcimb.2021.777070 PMC8913498 35282595

[31] Bao J Li C Zhang K Kang H Chen W Gu B. Comparative analysis of laboratory indexes of severe and non-severe patients infected with COVID-19. Clin Chim Acta. (2020) 509:180–94. doi: 10.1016/j.cca.2020.06.009, PMID: 32511971 PMC7274996

[32] Liu J Liu Y Xiang P Pu L Xiong H Li C. Neutrophil-to-lymphocyte ratio predicts critical illness patients with 2019 coronavirus disease in the early stage. J Transl Med. (2020) 18:1–12. doi: 10.1186/s12967-020-02374-0 32434518 PMC7237880

[33] Toori KU Qureshi MA Chaudhry A Safdar MF. Neutrophil to lymphocyte ratio (NLR) in COVID-19: A cheap prognostic marker in a resource constraint setting. Pak J Med Sci. (2021) 37:1435–9. doi: 10.12669/pjms.37.5.4194 34475926 PMC8377926

[34] Zuo Y Zuo M Yalavarthi S Gockman K Madison JA Shi H. Neutrophil extracellular traps and thrombosis in COVID-19. J Thromb Thrombolysis. (2021) 51:446–53. doi: 10.1007/s11239-020-02324-z, PMID: 33151461 PMC7642240

[35] Calabrese F Pezzuto F Fortarezza F Hofman P Kern I Panizo A. Pulmonary pathology and COVID-19: lessons from autopsy. The experience of European Pulmonary Pathologists. Virchows Arch. (2020) 477:359–72. doi: 10.1007/s00428-020-02886-6 32642842 PMC7343579

[36] Henry BM Benoit JL Benoit S Pulvino C Berger BA De OMHS. Red blood cell distribution width (RDW) predicts COVID-19 severity: a prospective, observational study from the Cincinnati SARS-CoV-2 Emergency Department Cohort. Diagnostics (Basel). (2020) 10:618. doi: 10.3390/diagnostics10090618, PMID: 32825629 PMC7554711

[37] Kiss S Gede N Hegyi P Németh D Földi M Dembrovszky F. Early changes in laboratory parameters are predictors of mortality and ICU admission in patients with COVID-19: a systematic review and meta-analysis. Med Microbiol Immunol. (2021) 210:33–47. doi: 10.1007/s00430-020-00696-w, PMID: 33219397 PMC7679241

[38] Wijarnpreecha K Ungprasert P Panjawatanan P Harnois DM Zaver HB Ahmed A. COVID-19 and liver injury: a meta-analysis. Eur J Gastroenterol Hepatol. (2021) 33:990–5. doi: 10.1097/MEG.0000000000001817, PMID: 32639420 PMC8162043

[39] Gracia-Ramos AE Jaquez-Quintana JO Contreras-Omaña R Auron M. Liver dysfunction and SARS-CoV-2 infection. World J Gastroenterol. (2021) 27:3951–70. doi: 10.3748/wjg.v27.i26.3951 34326607 PMC8311530

[40] Wanner N Andrieux G Badia-i-Mompel P Edler C Pfefferle S Lindenmeyer MT. Molecular consequences of SARS-CoV-2 liver tropism. Nat Metab. (2022) 4:310–9. doi: 10.1038/s42255-022-00552-6, PMID: 35347318 PMC8964418

[41] Qu J Zhu H-H Huang X-J He G-F Liu J-Y Huang J-J. Abnormal indexes of liver and kidney injury markers predict severity in COVID-19 patients. Infect Drug Resist. (2021) 14:3029–40. doi: 10.2147/IDR.S321915, PMID: 34408447 PMC8364353

[42] Ahmadian E Hosseiniyan Khatibi SM Razi Soofiyani S Abediazar S Shoja MM Ardalan M. Covid-19 and kidney injury: Pathophysiology and molecular mechanisms. Rev Med Virol. (2021) 31:e2176. doi: 10.1002/rmv.2176, PMID: 33022818 PMC7646060

[43] Stein C Nassereldine H Sorensen RJD Amlag JO Bisignano C Byrne S. Past SARS-CoV-2 infection protection against re-infection: a systematic review and meta-analysis. Lancet. (2023) 401:833–42. doi: 10.1016/S0140-6736(22)02465-5, PMID: 36930674 PMC9998097

[44] Bošnjak B Stein SC Willenzon S Cordes AK Puppe W Bernhardt G. Low serum neutralizing anti-SARS-CoV-2 S antibody levels in mildly affected COVID-19 convalescent patients revealed by two different detection methods. Cell Mol Immunol. (2021) 18:936–44. doi: 10.1038/s41423-020-00573-9, PMID: 33139905 PMC7604543

[45] Zhang B Yue D Wang Y Wang F Wu S Hou H. The dynamics of immune response in COVID-19 patients with different illness severity. J Med Virol. (2021) 93:1070–7. doi: 10.1002/jmv.26504 32910461

[46] Li T Wang X Zhuang X Wang H Li A Huang L. Baseline characteristics and changes of biomarkers in disease course predict prognosis of patients with COVID-19. Intern Emerg Med. (2021) 16:1165–72. doi: 10.1007/s11739-020-02560-4, PMID: 33565034 PMC7872821

[47] Zhang J Wang X Jia X Li J Hu K Chen G. Risk factors for disease severity, unimprovement, and mortality in COVID-19 patients in Wuhan, China. Clin Microbiol Infect. (2020) 26:767–72. doi: 10.1016/j.cmi.2020.04.012, PMID: 32304745 PMC7159868

