# Supplementary material for: Baseline and early changes in laboratory parameters predict disease severity and fatal outcomes in COVID-19 patients
Source: Front Public Health. 2023 Dec 13;11:1252358. doi: 10.3389/fpubh.2023.1252358 (PMC10751315; doi:10.3389/fpubh.2023.1252358)
Supplement: Supplementary file 1 [file Table_1.DOCX]

**Supplementary Table 1:- Comparison of COVID-19 fatal outcome in different comorbidities**

| Comorbidities | Outcome | | χ2 | P-Value |
| --- | --- | --- | --- | --- |
|  | Alive | Dead |  |  |
| Hypertension | 42 (87.5) | 6 (12.5) | 16.611 | 0.1648 |
| Diabetes | 20 (86.9) | 3 (13.1) |  |  |
| Diabetes and Hypertension | 19 (90.5) | 2 (9.5) |  |  |
| COPD | 2 (100) | 0 (0) |  |  |
| Respiratory Viral Infection | 7 (77.7) | 2 (22.3) |  |  |
| Crohn's disease | 3 (42.8) | 4 (57.2) |  |  |
| Acute respiratory distress syndrome | 4 (66.6) | 2 (33.4) |  |  |
| Asthma | 3 (60) | 2 (40) |  |  |
| Cardiac and Renal disease | 0 (0) | 1 (100) |  |  |
| Hypertension and Renal disease | 1 (50) | 1 (50) |  |  |
| Hypertension and Cardiac disease | 2 (100) | 0 (0) |  |  |
| Respiratory Viral Infection and MDD | 1 (100) | 0 (0) |  |  |
| Two or more of the above Disease | 13 (72.2) | 5 (27.8) |  |  |

*MDD (Major Depressive Disorder) COPD (Chronic obstructive pulmonary disease)
