# Supplementary material for: Baseline and early changes in laboratory parameters predict disease severity and fatal outcomes in COVID-19 patients
Source: Front Public Health. 2023 Dec 13;11:1252358. doi: 10.3389/fpubh.2023.1252358 (PMC10751315; doi:10.3389/fpubh.2023.1252358)
Supplement: Supplementary file 2 [file Table_2.DOCX]

**Supplementary Table 2:- Comparison of Age and final outcome in the presence and absence of comorbidity**

| **Comorbidity** | **Outcome** | **Age, X(IQR)** | **χ2** | **P- Value** |
| --- | --- | --- | --- | --- |
| No | Alive | 51 (38 - 60) | 723 | 0.000812 |
|  | Dead | 60 (56 - 70) |  |  |
| Yes | Alive | 57.5 (47.2 - 67.5) | 1226 | 0.2596 |
|  | Dead | 61 ( 50 - 72.2) |  |  |
