# Supplementary figures and images for: Baseline and early changes in laboratory parameters predict disease severity and fatal outcomes in COVID-19 patients
Source: Front Public Health. 2023 Dec 13;11:1252358. doi: 10.3389/fpubh.2023.1252358 (PMC10751315; doi:10.3389/fpubh.2023.1252358)

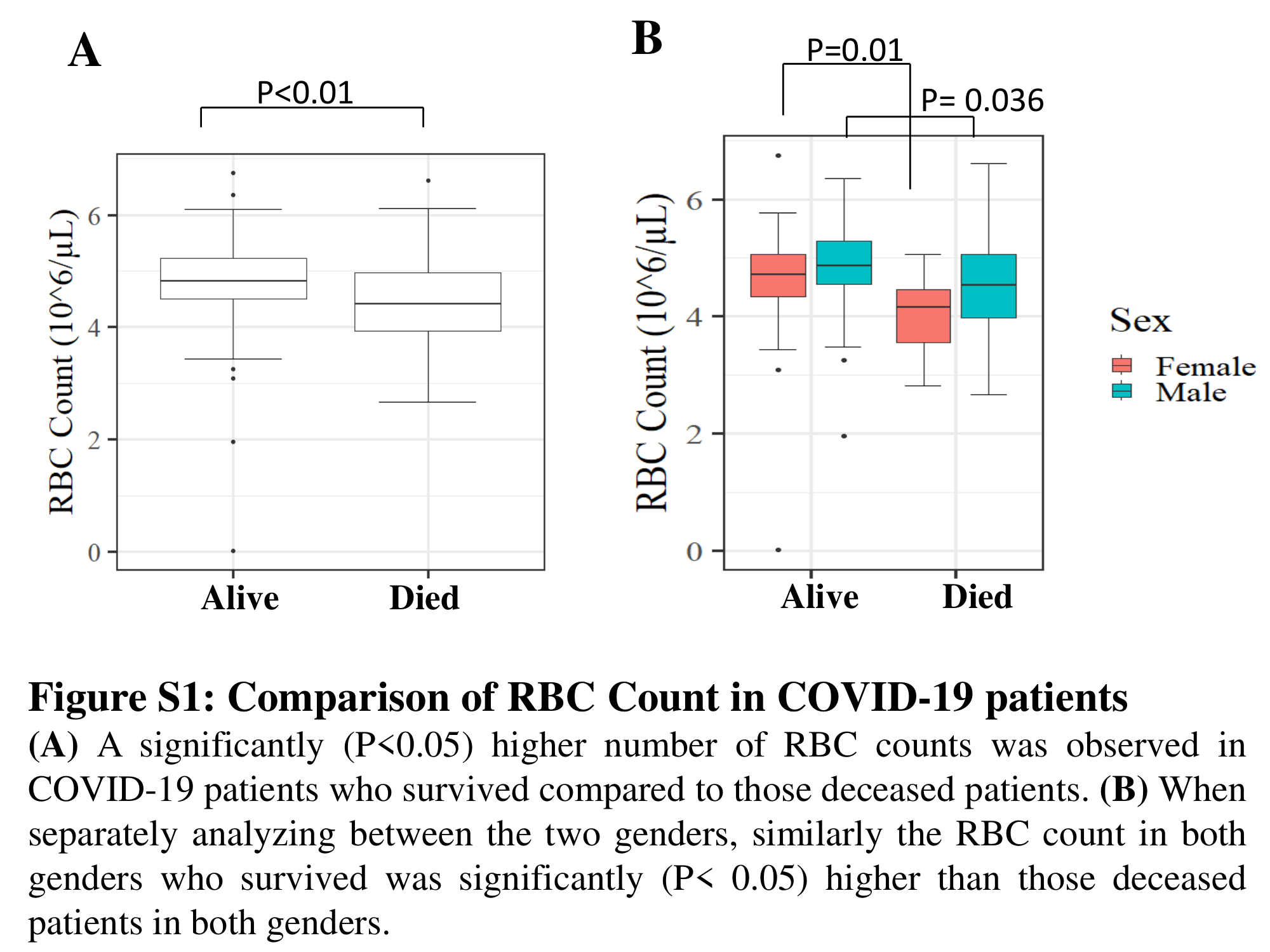

Supplement: Supplementary file 3 [file Image_1.TIFF]

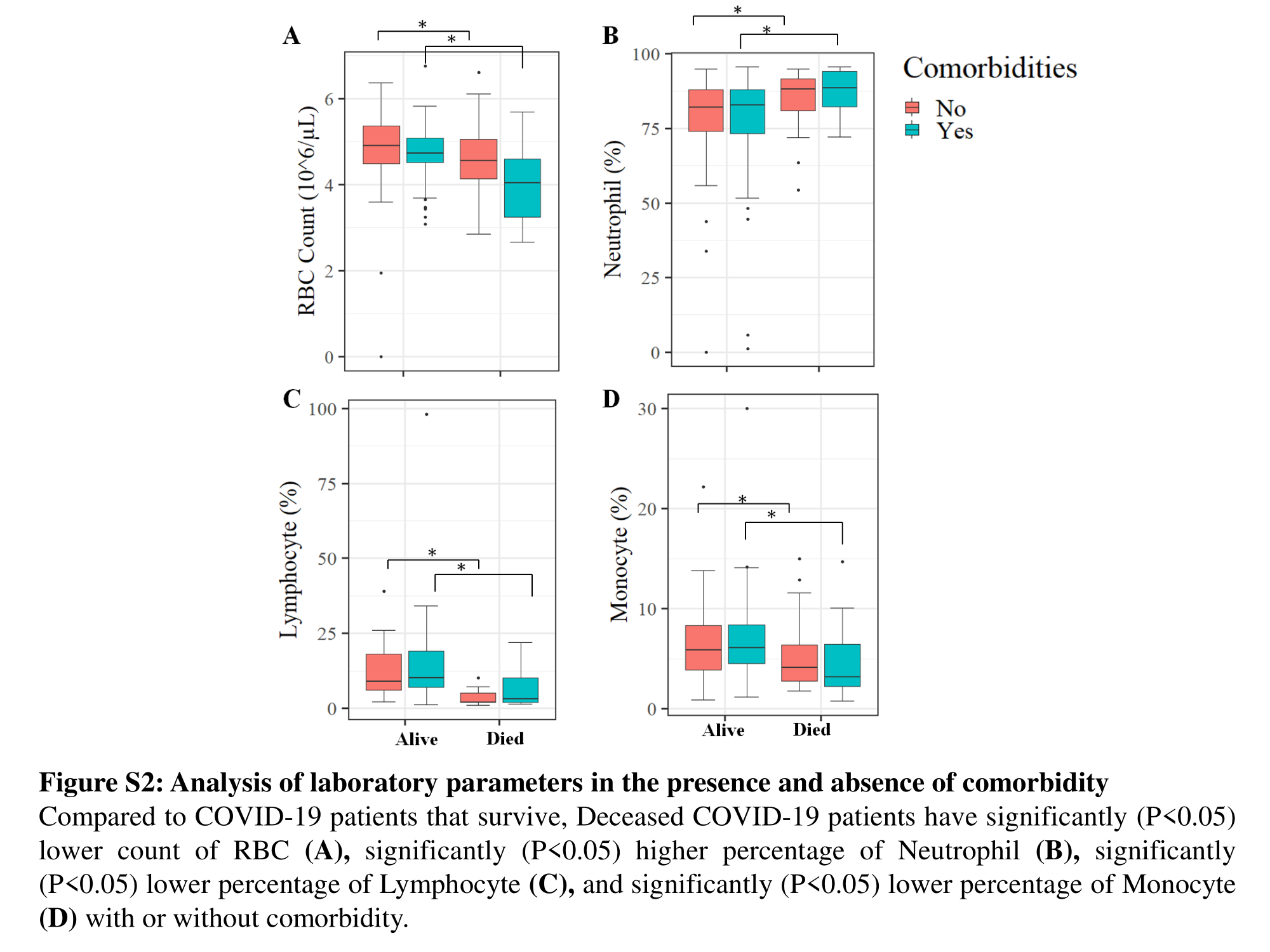

Supplement: Supplementary file 4 [file Image_2.TIFF]
